# Severe gonadotoxic insult manifests early in young girls treated for Ewing sarcoma

**DOI:** 10.1097/MD.0000000000004512

**Published:** 2016-08-19

**Authors:** Helena Mörse, Maria Elfving, Aleksandra Turkiewicz, Claus Yding Andersen, Ingrid Øra

**Affiliations:** a Department of Pediatrics, Pediatric Oncology and Hematology; b Department of Pediatrics, Pediatric Endocrinology; c Department of Orthopedics, Clinical Epidemiology Unit, Skane University Hospital, Lund University, Lund, Sweden; d Laboratory of Reproductive Biology, Rigshospitalet, University of Copenhagen, Copenhagen, Denmark.

**Keywords:** anti-Müllerian hormone, Ewing sarcoma, female fertility, osteosarcoma, ovarian reserve, Wilms tumor

## Abstract

We prospectively investigated anti-Müllerian hormone (AMH) as a measure of ovarian insult in young females during and after treatment for Wilms tumor (WT), osteosarcoma (OS), and Ewing sarcoma (ES).

Twenty-one female childhood cancer patients, with a mean age of 7.9 years (range 0.6–17), entered the study. Levels of AMH, follicle-stimulating hormone (FSH), and luteinizing hormone were monitored at diagnosis and every 3 to 4 months during, and regularly for a mean of 2.6 years after treatment.

A profound decline in AMH was seen in the majority of the 21 study patients 3 to 4 months after the beginning of treatment, the exception being patients with WT, of whom 60% showed no such decline. During the remaining treatment, all patients except those with WT not treated with whole abdominal radiotherapy or stem cell transplantation (SCT) had AMH below detection limit.

After completion of treatment, patients with OS and WT (without whole abdominal radiotherapy and SCT) recovered in AMH and had FSH in the normal range. In contrast, ES patients showed no AMH recovery and highly fluctuating FSH in the first years of follow-up, except for the 2 youngest patients, who had a late, slow AMH recovery.

In conclusion, young female ES patients already showed signs of severe ovarian dysfunction during the first years after cancer treatment similar to patients treated with SCT and abdominal radiotherapy, in contrast to females with WT and OS. Fertility counseling and information concerning fertility preservation procedures should be considered before starting to treat young females with ES.

## 1 Introduction

Focusing on potential long-term sequelae after pediatric cancer treatment is inevitable as the numbers and age of cancer survivors increase. Recent studies suggest that late effects might be experienced by approximately two-thirds of childhood cancer survivors.^[1]^ In female cancer survivors, ovarian dysfunction presents several clinical challenges, such as delayed or absent puberty, infertility, and primary ovarian insufficiency (POI).^[2]^ POI, defined as cessation of menses, low estrogen level, and increased gonadotropin levels before the age of 40, can develop during treatment, shortly afterward, or over time. In addition, women with low estrogen are at risk for osteoporosis, cardiovascular disease, and metabolic syndrome.^[3]^ The risk of POI increases in female cancer survivors treated with high doses of alkylating agents and pelvic radiation, as shown in several late effect studies.^[4]^ The exact cumulative toxic dose of chemotherapeutic agents that compromises ovarian function remains undefined.

Because anti-Müllerian hormone (AMH) correlates well with antral follicle count,^[5,6]^ it is now routinely used as a marker of ovarian reserve reflecting the primordial follicle pool.^[7–9]^ As AMH fluctuates only slightly during the menstrual cycle, measurements can be made regardless of the cycle phase in contrast to follicle-stimulating hormone (FSH).^[10]^ AMH in females, which peaks at the age of 24 years, can be detected from the end of gestation and levels increase during childhood and puberty. After a steady decline, it is no longer measurable at the age of menopause, approximately 50 to 51 years of age.^[11]^

The few prospective studies in girls and young women that measured AMH during and after treatment for cancer showed a rapid decline in AMH already after the start of cancer treatment, regardless of the therapy given.^[12–14]^ A study on 22 prepubertal and pubertal girls demonstrated that AMH was significantly reduced during treatment. The patients were divided into risk groups according to treatment; those in the high-risk group had undetectable AMH levels at the end of treatment and at the 6-month follow-up, as opposed to those in the medium- and low-risk groups.^[13]^

We observed a profound reduction in AMH 3 months after the start of cytotoxic treatment in our study of 34 pediatric female cancer patients before and after menarche. Girls with acute lymphoblastic leukemia recovered in AMH during maintenance treatment, whereas AMH levels in girls who received pelvic radiation or stem cell transplantation (SCT) remained undetectable at a median follow-up time of 18 months.^[12]^ A study on adult patients reported a decline in AMH after just 1 week of treatment for breast cancer, Ewing sarcoma (ES), and lymphoma.^[14]^

Patients with ES are treated with high doses of alkylating agents and are thereby at risk of POI and/or infertility, but whether the insult is acute or occurs over time is unknown. In this prospective single-center study, we investigated ovarian function in young females with ES and osteosarcoma (OS) during, and the first years after, treatment to study the dynamics of ovarian insult from cancer treatment, and compared them with a group of Wilms tumor (WT) patients with an expected low risk of ovarian insult.

## 2 Material and methods

### 2.1 Patients

Eligible patients were recruited from a cohort of 91 female cancer patients (0–18 years) who were followed for ovarian markers during and after treatment at the Department of Pediatric Oncology and Hematology, Skåne University Hospital, Lund, Sweden. From this population, 21 patients diagnosed with WT (10), OS (4), and ES (7) were enrolled in the present study. The clinical characteristics are shown in Table 1. The mean age at diagnosis for all patients was 7.9 years (range 0.6–17). In the WT group, mean age was 3.9 years, and for patients with OS and ES, 11.8 years and 11.2 years, respectively. Fifteen patients were premenarchal, as shown in Table 1. Of the 6 patients with menarche, none of them was on oral contraceptives at the time of diagnosis. Three resumed menstrual cycles during follow-up.

**Table 1:**
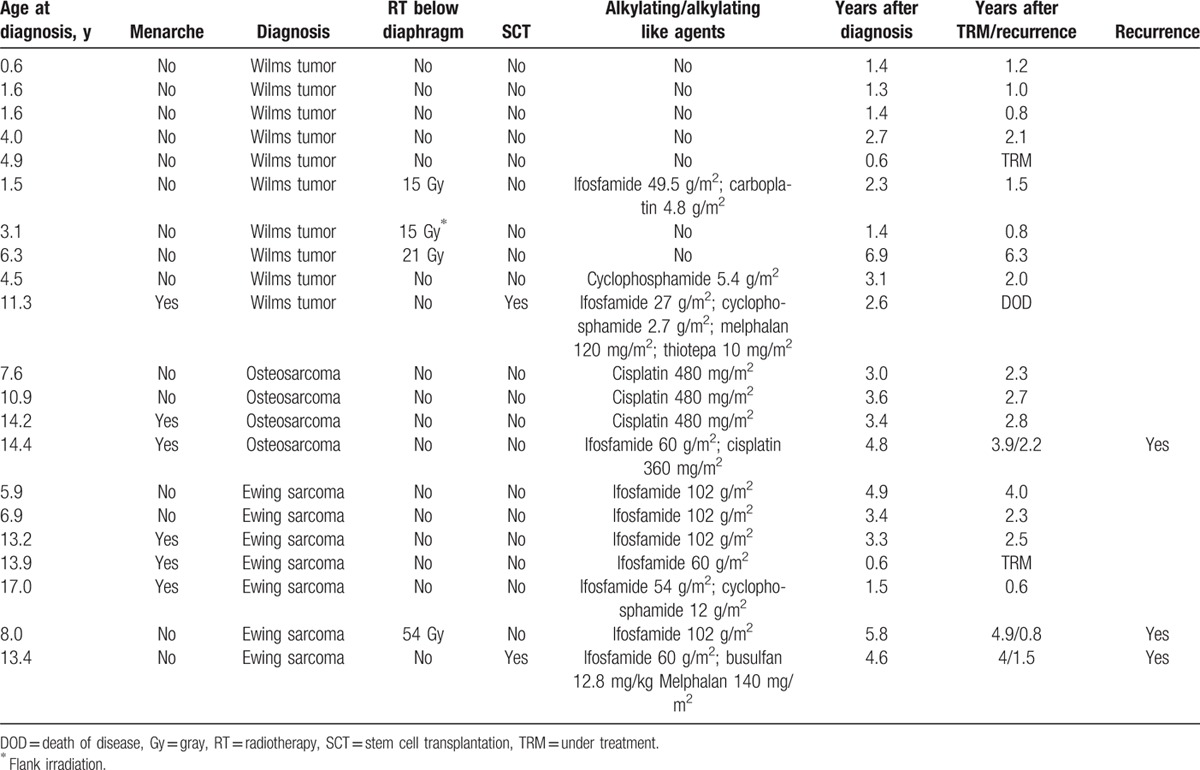
Clinical characteristics (n = 21).

The treatment regimens were administered according to national practice in Sweden. All patients were treated with chemotherapy. In addition, 3 received radiotherapy to the abdomen, and one had flank irradiation. Two patients underwent SCT (Table 1). Two patients are still undergoing treatment, and one died of disease.

### 2.2 Methods

Blood from a central venous catheter was sampled at diagnosis, approximately every 3 months during treatment, and 1 to 2 times yearly after treatment. The samples were centrifuged and aliquoted to assess AMH, FSH, and luteinizing hormone (LH) and were stored at −80°C until analysis. A total of 198 blood samples from unique time points were measured. During this longitudinal study, we experienced a freezer error with no measurable influence on the results.^[15]^

Serum levels of AMH were measured at the Laboratory of Reproductive Biology, Copenhagen, Denmark, using specific enzyme-linked immunosorbent assay (ELISA) kits according to the manufacturer's instructions (ultrasensitive AMH ELISA, AL-105-i Ansh Labs, Houston, TX). The detection level for AMH was 0.023 ng/mL. Plasma/serum levels of FSH (Roche 11775863) and LH (Roche 11732234) were measured at the Department of Clinical Chemistry, Skåne University Hospital Lund, Sweden. The detection limit in both assays was 0.2 IU/L.

Intra- and interassay coefficients of variation were <5%. All analyses were performed on thawed serum/plasma samples.

Written informed consent was obtained from each patient and/or her parents before inclusion in the study. The study was approved by the Regional Ethics Committee, Medical Faculty, Lund University, Sweden, approval number 211/2006.

### 2.3 Statistical analyses

All statistical analyses were performed using STATA version 13 (StataCorp LP, TX).

We used linear regression to analyze AMH levels, adjusting for age and diagnosis. We considered *P* < 0.05 statistically significant.

## 3 Results

### 3.1 Ovarian markers before treatment

Of the 20 patients with a pretreatment sample, all had detectable AMH levels, median 1.66 ng/mL (range 0.28–8.19), mean 1.93 ng/mL. Median pretreatment AMH for WT patients, OS, and ES was 1.29 ng/mL (range 0.34–3.42), 3.57 ng/mL (range 1.44–8.19), and 1.79 ng/mL (range 0.28–2.11), respectively. The pretreatment AMH levels were not statistically significantly higher in older patients or in those with menarche, *P* *=* 0.12 and 0.9, respectively.

After adjustment for age, patients with ES had statistically significantly lower AMH at diagnosis than patients with OS, β = −2.7, 95% confidence intervals (CIs) −4.8 to −0.6, *P* = 0.013. Those with WT also had lower values, although they were not statistically significant, β = −2.5, 95% CI −5.0 to 0.1, *P* = 0.054.

Pretreatment FSH was measurable in 20 patients with a median baseline level of 1.4 IU/L. LH was detectable in 5/20 (25 %) patients at diagnosis.

### 3.2 Ovarian markers during and after treatment

The time from diagnosis to final follow-up for patients with WT, OS, and ES was a mean of 2.4, 3.4, and 3.7 years, respectively. The duration of follow-up after completion of treatment was a mean of 1.9, 2.9, and 3.1 years, respectively.

#### 3.2.1 WT

**Figure 1:**
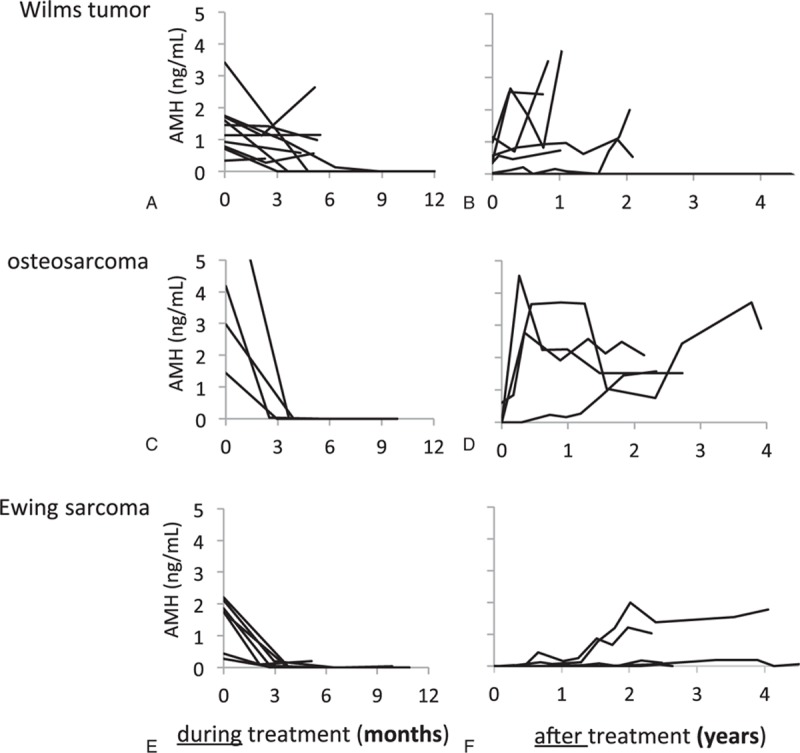
AMH levels before, during, and after cancer treatment of young female patients with Wilms tumor (A, B), osteosarcoma (C, D), and Ewing sarcoma (E, F). AMH = anti-Müllerian hormone.

Six of the 10 WT patients showed a minor or no decrease in AMH during treatment (Fig. 1A). Of the 4 patients with undetectable AMH during treatment, 3 treated with abdominal radiotherapy or SCT still had undetectable levels after treatment in contrast to the girl treated with flank irradiation, whose AMH recovered quickly.

**Figure 2:**
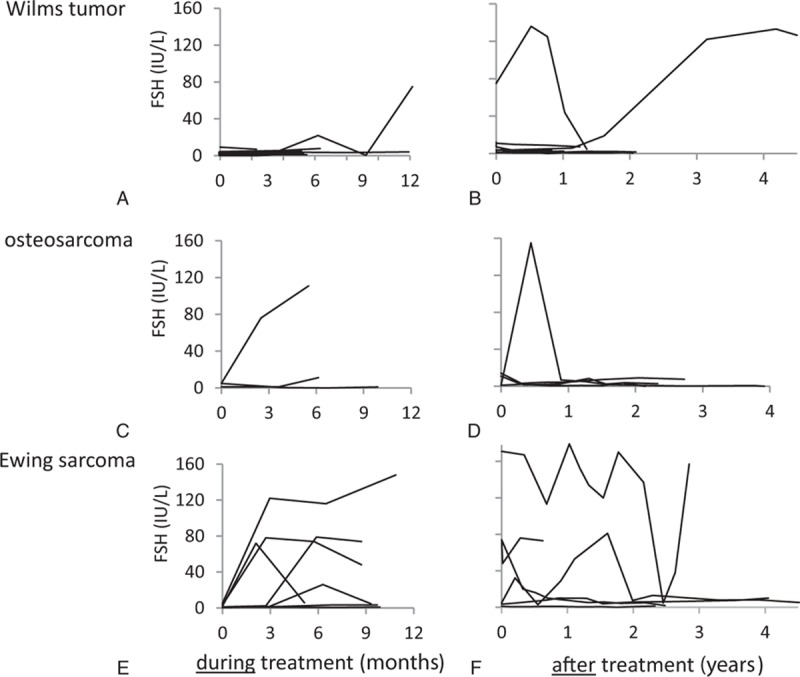
FSH levels before, during, and after cancer treatment of young female patients with Wilms tumor (A, B), osteosarcoma (C, D), and Ewing sarcoma (E, F). FSH = follicle-stimulating hormone.

Six patients showed increased AMH levels after treatment (Fig. 1B). FSH was in the normal range during and after treatment in all but 2 patients, as shown in Figure 2A, B. One girl with low FSH during treatment showed an increase to menopausal levels during follow-up 4 years after treatment ended, which was consistent with her having undetectable AMH. LH followed the same pattern as FSH (data not shown).

#### 3.2.2 OS

The patients with OS had undetectable or very low AMH during treatment, but they all recovered in AMH during follow-up (Fig. 1C, D). FSH increased in 1 girl during treatment, but returned to its normal level during follow-up (Fig. 2C, D). FSH was elevated in another patient at one measurement shortly after the end of treatment, but remained low at the 4-year follow-up (Fig. 2D). LH followed the pattern of FSH (data not shown).

#### 3.2.3 ES

All patients with ES had undetectable or very low AMH after 3 months and throughout treatment, as shown in Figure 1E. After treatment, 2 patients showed a late, slow increase in AMH (Fig. 1F). Interestingly, they were the 2 youngest patients, 5.9 and 7 years of age at diagnosis. FSH increased dramatically in 5 of the 7 girls with ES during treatment and fluctuated during follow-up (Fig. 2E). In 4 patients, FSH had returned to baseline at 2-year follow-up (Fig. 2F). LH followed the pattern of FSH (data not shown).

### 3.3 Comparison of AMH before treatment and at final follow-up

**Figure 3:**
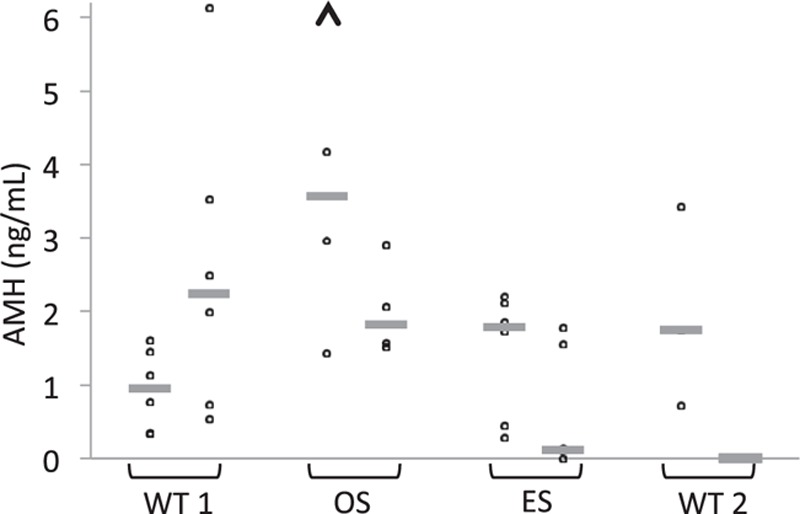
AMH levels at diagnosis and at final follow-up of young female patients after treatment for Wilms tumor (WT) without whole abdominal radiotherapy or stem cell transplantation (WT1), osteosarcoma (OS), Ewing sarcoma (ES), and WT including whole abdominal radiotherapy or stem cell transplantation (WT2). Time from diagnosis to final follow-up after completion of treatment was a mean of 1.5, 3.7, 3.9, and 3.9 years for the 4 groups, respectively. The arrowhead represents a value outside the diagram, 8.2. The gray lines depict the median AMH levels of each group. AMH = anti-Müllerian hormone.

Three patients with WT still had undetectable AMH at the final follow-up. They were all treated with whole abdominal radiation therapy or SCT (Fig. 3, WT2). Five of the 6 patients who recovered during follow-up increased in AMH above pretreatment level (Fig. 3, WT1), including one treated with flank irradiation and one treated with cyclophosphamide.

AMH increased in all patients with OS during follow-up. Two patients reached levels at, or slightly above, pretreatment level, of which one was the only treated with ifosfamide in addition to cisplatin (Fig. 3, OS and Table 1).

Four patients with ES had AMH levels that were extremely low or below the detection limit at the final follow-up (Fig. 3, ES). Two patients showed a late, slow increase in AMH (Fig. 1F), of whom one regained her pretreatment AMH level at 2-year follow-up.

There was a tendency, although not statistically significant, toward higher AMH concentration in the final posttreatment sample in the OS group than in patients with ES or WT.

## 4 Discussion

Knowledge about the risk of impaired fertility after childhood cancer treatment has emerged from retrospective studies of cancer survivors, decades after treatment. The 3 factors most strongly correlated to the risk of infertility are treatment with alkylating agents, pelvic radiotherapy, and treatment after puberty.^[16]^ The present investigation is one of few prospective studies in young females to evaluate ovarian function during cancer treatment and in the first years after treatment has ended.

In a published interim analysis of the present study, we reported a profound decline in AMH in all female childhood cancer patients after 3 months regardless of treatment given (n = 34).^[12]^ Those with detectable AMH after 3 months of treatment had a diversity of diagnoses and corresponding treatment regimens. The only WT patient without gonadotoxic treatment belonged to that group. In the present study we confirm the findings of a pronounced fall in AMH during the initial months of treatment, except for 6 of 10 patients with WT (Fig. 1A). So, with a larger WT cohort, we show that patients with WTs without abdominal radiotherapy or SCT have detectable, yet low AMH levels during treatment. These results probably reflect the high intensity of sarcoma treatment compared with that for WT. For the remainder of the treatment, all patients with ES or OS showed very low or undetectable AMH. Levels of FSH and LH differed, with pathological values in the majority of ES patients.

We observed low AMH levels at diagnosis among girls with WT, which most likely reflects their lower age. We also found considerably lower pretreatment AMH in patients with ES compared with patients with OS, which was not explained by a difference in age. Age distribution was similar in both sarcoma groups. Previous studies have shown reduced AMH levels at cancer diagnosis (before treatment) in girls ^[17]^ and women,^[18]^ suggesting that other factors, such as impaired general health status, might influence hormone levels. ES is considered a systemic disease,^[19,20]^ and studies report that one-third of patients have fever at diagnosis.^[21]^ Thus, it might be that ES in itself exerts a negative effect on the ovarian reserve.

In a German follow-up of both female and male patients treated for childhood cancer, the risk of infertility was assessed as 43% after ES and 31% after OS.^[16]^ Our cohort of female OS patients showed a prompt increase in AMH during the first years after treatment, whereas FSH remained low. These data probably reflect the fact that these young females have preserved ovarian function during the years immediately after treatment in contrast to the ES patients, although the potential risk of POI among them is unknown.

Alkylating agents included in sarcoma treatment protocols, such as ifosfamide and cyclophosphamide, are known to be gonadotoxic in a dose-dependent manner.^[2]^ The young females with ES were all treated with alkylating agents, and one also received pelvic radiotherapy at a dose of 54 Gy. We observed that the majority of the patients, in contrast to the patients with OS, had undetectable or very low AHM and high, fluctuating FSH during the first years following completion of treatment. These results correspond to a recent retrospective study in which 67% of young ES survivors had POI at a median follow-up of 5.7 years from diagnosis, with clinical signs of premature menopause in 37%.^[22]^

Similarly, female ES survivors in the Childhood Cancer Survival Study exhibited lower fertility rates with 29.7% among survivors and 40.1% in sibling controls. Interestingly, the likelihood of pregnancy was higher in those diagnosed at a younger age.^[23]^ Others have shown that older age at exposure to gonadotoxic treatment was found to be a risk factor for lower AMH levels in female childhood cancer survivors.^[24]^ We observed that the 2 youngest patients, 5.9 and 7 years of age, were the only ones to show a slow increase in AMH almost 2 years after completion of chemotherapy (Fig. 1F), which might reflect the results from the survivor studies above. In the present study, the patients with no AMH recovery at follow-up years after treatment are patients with ES or those treated with whole abdominal radiotherapy or SCT.

Some studies report an ongoing decline in AMH several years after cancer treatment in adult females.^[25]^ For this reason it is important to measure AMH despite normal menstrual cycles in young female survivors of OS or other diagnoses, as they may have a diminished ovarian follicular reserve despite having FSH and LH levels within the normal range. This time interval could be a window of intervention for fertility preservation procedures, such as ovarian hyperstimulation and cryopreservation of oocytes. Such procedures might also be an option upfront for young postpubertal females with an expected high risk of gonadal toxicity.

Upfront ovarian cryopreservation is an option for both pre- and postpubertal females with an estimated very high risk of gonadal toxicity.^[26]^ Today, approximately 80 live births are reported after autotransplantation of cryopreserved tissue (CY Andersen, personal communication). In 2015 the first live birth after prepubertal ovarian cryopreservation leading to pregnancy was reported.^[27]^ Reimplantation of ovarian tissue to ES patients remains a matter of debate due to the possible risk of transferring malignant cells in the ovarian tissue and the attendant risk of recurrence. Studies on in vitro oocyte maturation from cryopreserved ovarian tissue are ongoing; this procedure might be an opportunity for ES patients in the future.

As the small study population constitutes a limitation in the present study, we can only point to tendencies. More advanced statistical analyses with more factors taken into account were not feasible due to the relatively low number of patients. The large normal interindividual variation in AMH and the rise in AMH during childhood should also be considered. Another limitation might be the relatively short follow-up time, suggesting the need for caution when extrapolating to long-term outcomes. Nevertheless, the results presented here indicate a clear difference between the 3 different diagnostic groups.

In conclusion, young females with ES are at high risk of severe ovarian dysfunction during treatment and the first years afterward, in contrast to patients with OS and WT not treated with whole abdominal radiotherapy or SCT. Fertility counseling and information concerning fertility preservation procedures should be considered in ES patients before commencing cancer treatment.

## Acknowledgments

Research Nurse Ingrid Hagelin is gratefully acknowledged for continuous and skillful assistance and co-administration of the study as is Dr Pål W⊘lner-Hanssen for important steps in the initiation of the study.
